# Reproductive hormonal variations and adenohypophyseal lesions in pre-pubertal buffalo heifers inoculated with *Pasteurella multocida* type B: 2 and its immunogens

**DOI:** 10.1186/s12917-017-1010-y

**Published:** 2017-04-05

**Authors:** Faez Firdaus Abdullah Jesse, Hayder Hamzah Ibrahim, Yusuf Abba, Eric Lim Teik Chung, Ali Dhiaa Marza, Mazlina Mazlan, Mohd Zamri-Saad, Abdul Rahman Omar, Md Zuki Abu Bakar Zakaria, Abdul Aziz Saharee, Abd Wahid Haron, Mohd Azmi Mohd Lila

**Affiliations:** 1 Department of Veterinary Clinical Studies, Faculty of Veterinary Medicine, Universiti Putra Malaysia, 43400 Serdang, Malaysia; 2 Research Centre for Ruminant Diseases, Faculty of Veterinary Medicine, Universiti Putra Malaysia, 43400 Serdang, Malaysia; 3 BabilTechnical Institute, Al Furat Al-Awsat Technical University, Al-Hillah, Iraq; 4 Department of Veterinary Pathology and Microbiology, Faculty of Veterinary Medicine, Universiti Putra Malaysia, 43400 Serdang, Malaysia; 5 Department of Veterinary Internal Medicine, College of Veterinary Medicine, Al-Qasim Green University, Al-Qassim Buraidah, Iraq; 6 Institute of Bioscience, Universiti Putra Malaysia, 43400 Serdang, Malaysia

**Keywords:** Hemorrhagic septicemia, Reproductive hormones, Pituitary gland, Outer membrane protein, Lipopolysaccharide, Buffaloes

## Abstract

**Background:**

Hemorrhagic septicemia is a fatal disease of cattle and buffaloes caused by *P. multocida*. Although the pathogenesis of the bacteria has been well established in literature, there is a paucity of information on the possible role of the bacteria and its immunogens; lipopolysaccharide (LPS) and outer membrane proteins (OMPs) on the reproductive capacity of buffalo heifers.

**Methods:**

In this study, twenty one healthy prepubertal female buffaloes aged 8 months were divided into seven groups of 3 buffaloes each (G1-G7). Group 1 (G1) served as the negative control group and were inoculated orally with 10 mL sterile Phosphate Buffer Saline (PBS), groups 2 (G2) and 3 (G3) were inoculated orally and subcutaneously with 10 mL of 10^12^ colony forming unit (cfu) of *P.multocida* type B: 2, while groups 4 (G4) and 5 (G5) received 10 mL of bacterial LPS orally and intravenously, respectively. Lastly, groups 6 (G6) and 7 (G7) were orally and subcutaneously inoculated with 10 mL of bacterial OMPs. Whole blood was collected in EDTA vials at stipulated time points (0, 2, 4, 6, 8, 10, 12, 24, 36, 48, 72, 120, 168, 216, 264, 312, 360, 408, 456 and 504 h), while tissue sections of the pituitary glands were collected and transported to the histopathology laboratory in 10% buffered formalin for processing and Hematoxylin and eosin staining. Plasma levels of luteinizing hormone (LH), follicle stimulating hormone (FSH), progesterone (PG), estradiol (EST) and gonadotrophin releasing hormone (GnRH) were determined.

**Results:**

The histopathological lesions observed in the pituitary gland included hemorrhage, congestion, inflammatory cell infiltration, hydropic degeneration, necrosis and edema. These changes were higher (*p* < 0.05) in distribution and severity in G3, G6 and G7. Hormonal concentrations of LH, FSH, PG, EST and GnRH declined in all inoculation groups as time elapsed and were lower (*p* < 0.05) than that of the control group.

**Conclusion:**

Based on these findings, *P.multocida* B: 2 and its immunogens can be said to negatively affect the hypothalamic-pituitary-gonadal axis, resulting in decreased levels of reproductive hormones which may predispose to infertility in buffalo heifers.

## Background

Haemorrhagic Septicaemia (HS) is an important disease of cattle and buffaloes caused by *Pasteurella multocida* [1]. It can be described as an acute, fatal septicemic disease caused by specific serotypes of *Pasteurella multocida*. *P. multocida* contains lipopolysaccharide (LPS), which is an important component of the outer cell wall of the organism [2–4]. The LPS are released during bacterial multiplication or death, resulting in an inflammatory reaction in the host animal. The LPS is part of the bacterial endotoxin and plays an important role in the pathogenesis of the disease [5, 6]. Another immunogen of *P. multocida* is a 37 kd outer membrane proteins (OMPs), which is one of the most important virulence factors of *Pasteurella multocida* type B: 2. The components of the bacterial outer membrane such as trans membrane proteins and lipoproteins play key roles in the interaction of the pathogen with the host environment and in the host immune response to infection [7]. Both the LPS and OMPs showed to induce immunogenic responses in laboratory animals and hence useful in the development of immunogenic vaccines [5, 8].

In our previous studies, both *P. multocida* and its immunogen; LPS induced pathological lesions in the reproductive system and pituitary glands of both male and female mice. Furthermore, we also reported changes in the hormonal profiles of estrogen, progesterone and testosterone in the infected mice [9]. Recently, we also reported histopathological changes in the reproductive tract of buffalo heifers infected with *P. multocida* and its immunogen; LPS and OMPs [10, 11]. The studies above all showed the possibility of *P. multocida* and its immunogens to induce reproductive dysfunction, which may result in infertility in infected animals. However, since the pituitary gland and associated hormones play vital roles in the reproductive cycle of female animals, we hypothesized that *P. multocida* and its immunogen; LPS and OMPs will cause changes in reproductive hormonal levels and pathology in the pituitary gland of pre-pubertal female buffaloes.

## Methods

### Animal housing, grouping and inoculation

The animals were housed in pens with concrete floors. At the commencement of the experiment, the animals were divided into their respective inoculation groups and housed separately. Food and water were provided ad libatum. Twenty one healthy prepubertal female buffaloes aged 8 months were used for this study. Preparation of P*. multocida* type B: 2 culture was done as described previously [10], while the LPS and OMPs were extracted as previously described [11].

Briefly, LPS was extracted using Intron Biotechnology LPS extraction kit. 5 mL of the bacterial cell suspension containing 10 ^12^ cfu of *P. multocida* was centrifuged at 13, 000 rpm for 30 s at room temperature. Extraction was done as stated in the manufacturer’s protocol (http://eng.intronbio.com/PROTO-PDF/LPS%20Extraction%20Kit.pdf). SDS-PAGE was used to confirm the absence of protein in the extracted LPS.

The extraction of OMPs was carried out by freezing freshly harvested bacterial cell pellets for 24 h prior to extraction. The cell pellets were thawed for 15 min on ice and re-suspended in 10 mL of native lysis buffer. The cells were then incubated on ice for 30 min followed by centrifugation at 14,000 rpm for 30 min at 4 °C. The resultant supernatant containing the soluble fraction of the bacterial outer membrane proteins was retained and used in this study [11]. The female buffaloes were divided into seven groups of 3 buffaloes each (G1-G7). Group 1 (G1) served as the negative control group and were inoculated orally with 10 mL sterile Phosphate Buffer Saline (PBS), groups 2 (G2) and 3 (G3) were inoculated orally and subcutaneously with 10 mL of 10^12^ colony forming unit (cfu) of *P.multocida* type B: 2, while groups 4 (G4) and 5 (G5) received 10 mL of bacterial LPS orally and intravenously, respectively. Lastly, groups 6 (G6) and 7 (G7) were orally and subcutaneously inoculated with 10 mL of bacterial OMPs. The animals were monitored every hour for clinical signs or signs of distress.

### Exsanguination of animals

After 12 h of inoculation, calves from *P.multocida* subacute inoculation group were showing severe signs of respiratory distress and recumbency and had to be euthanized in order to minimize pain and suffering. Similarly, after 72 h, calves from the OMPs subacute inoculation group were also exhibiting severe signs of respiratory distress and recumbency and were also euthanized. All calves from the other groups survived to the end of the experimental period (21 days) and were euthanized at 21 days. Euthanasia was carried out by exsanguination after the animals were anesthetized with Xylazine (1 mg/kg).

### Sample collection and processing

Whole blood was collected in EDTA vials at stipulated time points (0, 2, 4, 6, 8, 10, 12, 24, 36, 48, 72, 120, 168, 216, 264, 312, 360, and 408, 456 and 504 h). The blood was centrifuged at 2, 400×g for 10 min to collect the plasma. Following the euthanasia of animals in G3 and G7, at 24 h and 72 h, and other animals at 21 days, the pituitary glands were grossly examined for changes and tissue samples of the pituitary glands were collected and transported to the histopathology laboratory in 40% buffered formalin. Fixed tissue samples were processed by serial dehydration in ethanol, embedding in paraffin, sectioning at 4 μm and staining with Hematoxylin and Eosin (H&E). Stained slides were examined under light microscopy at 200 X for lesions suggestive of infection such as hemorrhage, congestion, inflammatory cell infiltration, degeneration, necrosis and edema. From each tissue section, a total of 6–10 microscopic focal areas were evaluated and scored for lesion severity. Lesions were scored by using a grading scale of 0 (none), 1 (mild), 2 (moderate) and 3 (severe) based on the distribution in the microscope focus as previously described [12].

### Evaluation of reproductive hormonal levels

Radio immunoassay (RIA) Kits (Immunotech, Beckman Coulter, U.S.A) were used for the plasma detection of luteinizing hormone (LH), follicle stimulating hormone (FSH), progesterone (PG) and estradiol (EST), while gonadotrophin releasing hormone (GnRH) was determined with an ELISA kit (Qayee Bio, China). All protocols followed were based on the manufacturer’s instructions without any modifications. Briefly, in the RIA hormonal evaluation for LH and FSH, the plasma sample was added to 100 μL of calibrator which was mixed with 50 μL of tracer and added to antibody coated tubes. The tubes were covered and incubated for 90 min at 37 °C. Count bound per minute was determined using Wallac Wizard Gamma Counter model 1470. The determination of EST and PG were done using the methods previously described by Jesse et al. [13], while GnRH concentration was determined by preparing sample and standard as outlined in the manufacturer’s instructions. Briefly, both sample and standard were added with HRP-conjugate reagent and incubated for 60 min at 37 °C. The 96 well plate was washed five times and chromogen solution A,B was added. The plate was incubated for 10 min at 37 °C and the optical density was measured at 450 nm.

### Statistical analysis

Hormonal concentrations were summarized into mean and standard error of means and analysed using Graph Pad Prism (Version 6.0), with Kruskal Wallis test (non-parametric) with Dunn’s multiple comparison. Statistical significance was set at *p* < 0.05.

## Results

### Clinical findings

The clinical findings observed in the inoculation groups have been described in an earlier study by Ibrahim et al. [10, 11]. Briefly, fever was observed in all the groups for at least 3 days post inoculation. Respiratory distress and mucopurulent discharge were mild in groups G2, G4, G5 and G6, while groups G3 and G7 developed severe signs and had to be euthanized. The mucous membrane was normal in G2, G4 and G6, while slight congestion was observed in G5 and moderate to severe in G3 and G7.

### Gross and histopathological lesions in the pituitary glands

**Fig. 1:**
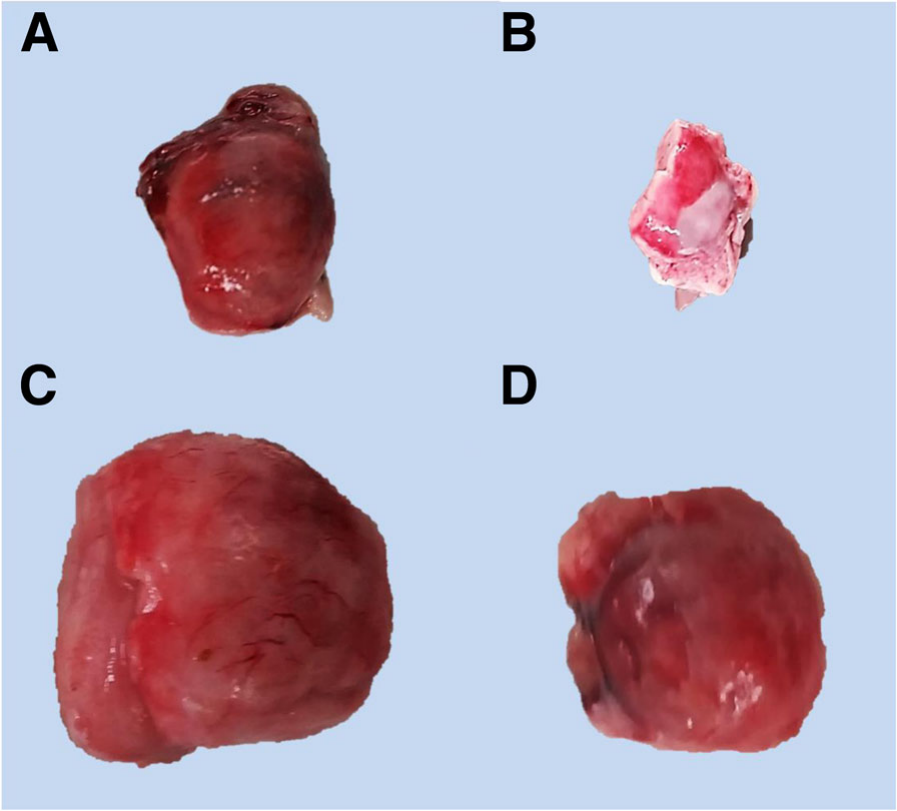
Photograph of the pituitary gland of pre-pubertal buffalo calves following (**a**) subcutaneous inoculation of *Pasteurella multocida* B: 2 showing moderate congestion (**b**) subcutaneous inoculation with bacterial OMPs showing moderate congestion (**c**) oral inoculation with *Pasteurella multocida* B:2 showing mild congestion (**d**) oral inoculation with bacterial OMPs showing moderate congestion

The most obvious gross change observed in pituitary gland was congestion, which was mild to moderate in G3, G6 and G7, and mild G2, G4 and G5. The negative control animals did not show any lesions at postmortem examination (Fig. 1).

**Fig. 2:**
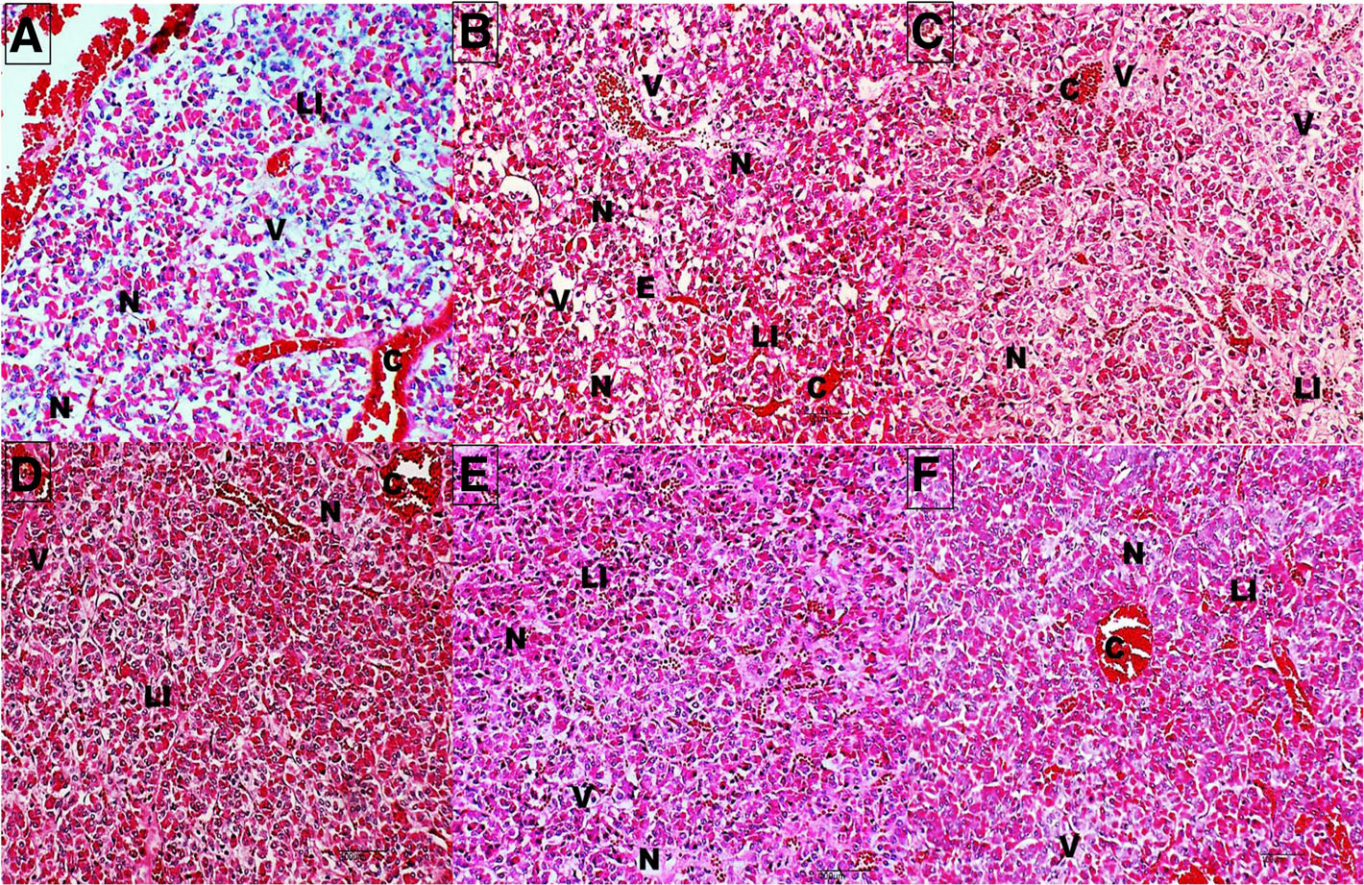
Photomicrograph section of the anterior pituitary gland from different inoculation groups (**a**) *P. multocida* type B:2 subcutaneous inoculation (**b**) OMPs subcutaneous inoculation (**c**) OMPs oral inoculation (**d**) LPS intravenous inoculation (**e**) LPS oral inoculation (**f**) *P. multocida* B:2 oral inoculation groups showing vascular congestion (**c**) and presence of leucocytic infiltration (LI) around cells (Basophils) undergoing hydropic/vacuolar degeneration (V) and necrosis (N), note also the presence of edema (**e**), H&E × 200

The histopathological lesions observed in the pituitary gland are summarized in Table 1. Hemorrhage and congestion were higher (*p* < 0.05) in G3 and G7.Inflammatory cell responses typified by infiltrations of neutrophils and lymphocyte was comparable in all groups (*p* > 0.05), but higher (*p* < 0.05) in G7. Hydropic degeneration characterized by presence of vacuoles were commonly observed in the basophils than in the acidophils. Necrosis was characterized by nuclear pyknosis and karyorrhexis and in some instances complete nuclear karyolysis. Both degeneration and necrosis were comparable and lower (*p* > 0.05) in G2, G4 and G5, and higher (*p* < 0.05) in G3, G6 and G7. Edema was comparable and higher in G3, G6 and G7 (Fig. 2).

**Table 1:** Mean score of cellular changes in the pituitary gland of Pre-pubertal Buffalo heifers inoculated with *Pasteurella multocida* B: 2 and its immunogens (LPS and OMPs)

Parameters					
Groups	Haemorrhage and Congestion	Inflammatory Cell Infiltration	Necrosis	Degeneration	Edema
Group 1 (PBS Control)	0.0 ± 0.0^a^	0.0 ± 0.0^a^	0.0 ± 0.0^a^	0.0 ± 0.0^a^	0.0 ± 0.0^a^
Group 2 (*P.multocida* B:2 Oral)	0.42 ± 0.11^b^	0.33 ± 0.10^a^	0.81 ± 0.12^b^	1.52 ± 0.10^b^	0.17 ± 0.08^a^
Group 3 (*P.multocida* B:2 Subcutaneous)	2.50 ± 0.18^c^	0.33 ± 0.10^a^	1.64 ± 0.13^c^	2.43 ± 0.15^c^	0.42 ± 0.11^b^
Group 4 (LPS Oral)	0.58 ± 0.16^b^	0.33 ± 0.16^a^	0.75 ± 0.15^b^	1.45 ± 0.12^b^	0.25 ± 0.14^a^
Group 5 (LPS Intravenous)	0.67 ± 0.16^b^	0.33 ± 0.16^a^	0.80 ± 0.11^b^	1.05 ± 0.15^b^	0.17 ± 0.11^a^
Group 6 (OMP Oral)	2.58 ± 0.18^b^	0.33 ± 0.14^a^	1.35 ± 0.15^c^	2.31 ± 0.15^c^	0.50 ± 0.17^b^
Group 7 (OMP Subcutaneous)	2.75 ± 0.18^c^	0.42 ± 0.14^b^	1.55 ± 0.10^c^	2.44 ± 0.12^c^	0.42 ± 0.11^b^

All values are expressed as mean ± SE; a, b, c values with superscript within columns are significantly different at *P* < 0.05

## Reproductive hormone analysis

### Gonadotrophin releasing hormone concentration

The changes in the mean concentration of GnRH in all groups are summarized in Table 2. The concentration of GnRH was lower (*p* < 0.05) in all the inoculation groups from 2 h of infection and kept declining as the time of inoculation progressed. After 12 h, GnRH was low (*p* < 0.05) in the G3. By 24, 36, 48, 60 and 72 h of inoculation, GnRH was lower (*p* < 0.05) in G7 when compared to other groups. After 504 h, GnRH concentration was lower (*p* < 0.05) in all inoculation groups, but comparable in G2, G5 and G6, while G4 had the lowest GnRH concentration.

**Table 2:** Changes in mean concentration of Gonadotropin-releasing hormone (GnRH) in prepubertal buffalo heifers following inoculation with *Pasteurella multocida* B: 2 and its immunogens (LPS and OMP)

Time (hr)	Inoculated with phosphate buffer saline	Inoculated with *P.multocida* B:2		Inoculated with LPS extracted from *P.multocida* B:2		Inoculated with OMPs extracted from *P.multocida* B:2	
	Control –G1	Oral route –G2	Subcute route - G3	Oral route- G4	Intravenous route – G5	Oral route- G 6	Subcute route - G7
0	218.5 ± 3.4^ab^	214.2 ± 0.1^ab^	218.2 ± 0.01^a^	221.6 ± 2.1^a^	212.8 ± 0.2^ab^	215.7 ± 0.1^ab^	205.7 ± 0.3^c^
2	222.6 ± 3.6^a^	169.1 ± 0.0^b^	155.4 ± 1.1^b^	207.5 ± 0.2^c^	201.6 ± 0.0^c^	186.1 ± 1.9^d^	154.9 ± 0.2^b^
4	222.2 ± 6.1^a^	152.7 ± 0.02^b^	155.4 ± 0.0^b^	186.2 ± 0.01^c^	181.6 ± 0.01^c^	173.8 ± 0.1^d^	157.2 ± 0.0^b^
6	217.4 ± 3.4^a^	148.6 ± 1.1^b^	140.1 ± 1.0^c^	170.7 ± 1.0^d^	167.5 ± 0.2^d^	169.2 ± 2.1^d^	139.5 ± 2.3^c^
8	210.9 ± 10.7^a^	144.9 ± 0.0^b^	111.9 ± 0.1^c^	164.1 ± 0.0^d^	167.9 ± 2.1^d^	165.5 ± 0.02^d^	120.9 ± 2.1^e^
10	209.9 ± 7.1^a^	143.1 ± 0.1^b^	86.5 ± 0.1^c^	158.4 ± 0.2^d^	147.7 ± 0.1^b^	158.7 ± 0.0^d^	119.6 ± 2.3^e^
12	212.2 ± 2.9^a^	130.8 ± 0.1^b^	82.9 ± 0.0^c^	151.5 ± 1.1^d^	145.1 ± 0.0^e^	158.3 ± 0.01^f^	101.0 ± 0.8^g^
24	217.4 ± 6.6^a^	138.7 ± 1.0^b^		148.0 ± 0.1^c^	140.7 ± 0.2^b^	147.4 ± 0.01^c^	121.7 ± 0.0^d^
36	217.9 ± 2.5^a^	127.3 ± 0.0^b^		137.7 ± 0.1^c^	130.4 ± 0.3^d^	134.6 ± 1.02^c^	107.6 ± 0.02^e^
48	215.1 ± 0.0^a^	114.3 ± 5.7^b^		129.7 ± 0.2^c^	122.8 ± 1.1^d^	126.8 ± 0.1^c^	105.9 ± 0.0^e^
60	218.5 ± 1.9^a^	111.8 ± 0.1^b^		113.8 ± 0.02^b^	119.3 ± 0.01^c^	108.3 ± 0.2^b^	78.3 ± 1.9^d^
72	220.0 ± 3.2^a^	96.3 ± 1.1^b^		110.2 ± 0.1^c^	113.9 ± 0.2^c^	110.1 ± 0.0^c^	88.1 ± 0.0^d^
120	214.9 ± 2.2^a^	94.3 ± 0.9^b^		109.9 ± 0.7^bd^	106.9 ± 0.3^b^	120.2 ± 1.1^bd^	
168	216.5 ± 8.5^a^	84.7 ± 4.9^b^		104.5 ± 0.6^cd^	102.9 ± 1.2^c^	112.6 ± 1.5^d^	
216	215.3 ± 5.8^a^	91.2 ± 0.0^b^		101.6 ± 1.2^c^	98.4 ± 0.01^c^	106.5 ± 1.2^d^	
264	218.9 ± 5.3^a^	89.3 ± 0.2^b^		100.4 ± 2.2^c^	95.3 ± 2.1^b^	100.6 ± 2.1^c^	
312	218.6 ± 3.6^a^	85.7 ± 2.3^b^		93.2 ± 0.01^c^	88.9 ± 1.1^b^	95.9 ± 0.2^c^	
360	218.6 ± 7.1^a^	78.8 ± 1.0^b^		85.3 ± 2.1^c^	83.2 ± 2.1^c^	99.9 ± 2.3^d^	
408	215.1 ± 0.1^a^	77.3 ± 1.9^b^		74.4 ± 0.02^b^	76.1 ± 0.2^c^	87.6 ± 4.7^c^	
456	216.5 ± 2.9^a^	75.3 ± 0.3^b^		62.1 ± 0.0^c^	74.3 ± 0.3^b^	76.7 ± 0.5^b^	
504	218.1 ± 4.8^a^	74.1 ± 0.1^b^		62.5 ± 0.02^c^	72.2 ± 0.9^b^	75.4 ± 0.7^b^	

All values are expressed as mean ± SE; a, b, c, d, e, f, g values with superscript within rows are significantly different at *P* < 0.05

### Follicle stimulating hormone concentration

The changes in the mean concentration of FSH in all groups are summarized in Table 3. There were no changes in FSH in all groups after 6 h, while at 12 h FSH was lower (*p* < 0.05) in G3 and by 48 h G7 had a lower FSH concentration among the inoculation groups. After 48 h, the concentration of FSH was comparable in all inoculation groups but lower (*p* < 0.05) than the control group. Similarly, the level of FSH declined gradually down the various inoculation groups as time elapsed.

**Table 3:** Changes in mean concentration of Follicle stimulating hormone in prepubertal buffalo heifers following inoculation with *Pasteurella multocida* B: 2 and its immunogens (LPS and OMP)

Time (hr)	Inoculated with PBS	Inoculated with *P.multocida* B:2		Inoculated with LPS extracted from *P.multocida* B:2		Inoculated with OMPs extracted from *P.multocida* B:2	
	Control –G1	Oral route –G2	Subcutaneous route - G3	Oral route- G4	Intravenous route – G5	Oral route- G 6	Subcutaneous route - G7
0	0.32 ± 0.01^a^	0.31 ± 0.00^a^	0.33 ± 0.01^a^	0.32 ± 0.01^a^	0.33 ± 0.01^a^	0.32 ± 0.00^a^	0.32 ± 0.00^a^
2	0.32 ± 0.01^ab^	0.30 ± 0.00^bc^	0.27 ± 0.01^c^	0.32 ± 0.00^ab^	0.33 ± 0.00^a^	0.30 ± 0.00^abc^	0.31 ± 0.01^abc^
4	0.31 ± 0.00^ab^	0.29 ± 0.00^bcd^	0.25 ± 0.02^d^	0.31 ± 0.00^a^	0.31 ± 0.01^abc^	0.29 ± 0.00^abc^	0.28 ± 0.01^cd^
6	0.30 ± 0.00^a^	0.28 ± 0.01^a^	0.24 ± 0.01^a^	0.30 ± 0.00^a^	0.29 ± 0.02^a^	0.29 ± 0.01^a^	0.27 ± 0.01^a^
8	0.33 ± 0.00^a^	0.28 ± 0.01^b^	0.23 ± 0.00^b^	0.29 ± 0.00^ab^	0.28 ± 0.01^ab^	0.28 ± 0.01^ab^	0.25 ± 0.02^b^
10	0.32 ± 0.00^a^	0.26 ± 0.01^b^	0.21 ± 0.01^b^	0.29 ± 0.01^ab^	0.27 ± 0.01^b^	0.27 ± 0.01^b^	0.24 ± 0.02^b^
12	0.33 ± 0.00^a^	0.26 ± 0.00^bc^	0.19 ± 0.00^d^	0.28 ± 0.01^b^	0.25 ± 0.01^bc^	0.26 ± 0.01^bc^	0.23 ± 0.00^c^
24	0.31 ± 0.00^a^	0.25 ± 0.02^b^		0.27 ± 0.01^b^	0.25 ± 0.01^b^	0.25 ± 0.01^b^	0.21 ± 0.01^b^
36	0.32 ± 0.0l^a^	0.24 ± 0.01^b^		0.27 ± 0.02^b^	0.24 ± 0.00^b^	0.23 ± 0.02^b^	0.20 ± 0.0^b^
48	0.31 ± 0.0l^a^	0.24 ± 0.01^bc^		0.26 ± 0.00^b^	0.23 ± 0.01^bc^	0.23 ± 0.01^bc^	0.20 ± 0.00^c^
60	0.31 ± 0.00^a^	0.23 ± 0.00^b^		0.24 ± 0.00^b^	0.22 ± 0.02^b^	0.21 ± 0.02^b^	0.19 ± 0.01^b^
72	0.33 ± 0.00^a^	0.23 ± 0.01^b^		0.22 ± 0.01^b^	0.22 ± 0.00^b^	0.21 ± 0.01^b^	0.18 ± 0.01^b^
120	0.32 ± 0.00^a^	0.21 ± 0.00^b^		0.22 ± 0.02^b^	0.21 ± 0.01^a^	0.2 ± 0.00^a^	
168	0.32 ± 0.00^a^	0.19 ± 0.00^b^		0.22 ± 0.00^b^	0.20 ± 0.00^b^	0.20 ± 0.01^b^	
216	0.31 ± 0.00a	0.18 ± 0.01^b^		0.19 ± 0.01b	0.20 ± 0.01^b^	0.19 ± 0.02^b^	
264	0.33 ± 0.00^a^	0.18 ± 0.01^b^		0.21 ± 0.01^b^	0.18 ± 0.02^b^	0.18 ± 0.02^b^	
312	0.32 ± 0.00^a^	0.17 ± 0.01^b^		0.21 ± 0.01b	0.18 ± 0.01b	0.19 ± 0.01^b^	
360	0.31 ± 0.00^a^	0.17 ± 0.01^b^		0.19 ± 0.003^b^	0.18 ± 0.02^b^	0.18 ± 0.01^b^	
408	0.30 ± 0.00^a^	0.16 ± 0.00^b^		0.19 ± 0.01^b^	0.18 ± 0.02^b^	0.18 ± 0.00^b^	
456	0.31 ± 0.03^a^	0.16 ± 0.01^b^		0.18 ± 0.01^b^	0.18 ± 0.00b	0.18 ± 0.01^b^	
504	0.32 ± 0.01^a^	0.16 ± 0.01^b^		0.17 ± 0.01^b^	0.17 ± 0.01^b^	0.16 ± 0.00^b^	

All values are expressed as mean ± SE; a, b, c, d, e, f, g values with superscript within rows are significantly different at *P* < 0.05

### Luteinizing hormone concentration

The changes in mean concentration of LH in all groups are summarized in Table 4. After 4 h of inoculation, G3, G4, G5 and G7 had the lowest (*p* < 0.05) concentration of LH. However, there were no changes among the inoculation groups during other sampling periods. Furthermore, the concentration of LH declined down the inoculation groups as time elapsed and were lower (*p* < 0.05) than the control group.

**Table 4:** Changes in mean concentration of Luteinizing hormone in prepubertal buffalo heifers following inoculation with *Pasteurella multocida* B: 2 and its immunogens (LPS and OMP)

Time (hr)	Inoculated with PBS	Inoculated with *P.multocida* B:2		Inoculated with LPS extracted from *P.multocida* B:2		Inoculated with OMPs extracted from *P.multocida* B:2	
	Control –G1	Oral route –G2	Subcutaneous route - G3	Oral route- G4	Intravenous route – G5	Oral route- G 6	Subcutaneous route - G7
0	0.51 ± 0.01^a^	0.52 ± 0.01 ^a^	0.53 ± 0.00^a^	0.49 ± 0.01^a^	0.48 ± 0.01^a^	0.52 ± 0.01^a^	0.51 ± 0.01^a^
2	0.50 ± 0.00^a^	0.43 ± 0.01^b^	0.43 ± 0.01^b^	0.48 ± 0.02^ab^	0.42 ± 0.01^b^	0.5 ± 0.01a	0.44 ± 0.01^b^
4	0.52 ± 0.01^a^	0.41 ± 0.00c	0.39 ± 0.01^c^	0.43 ± 0.01^c^	0.40 ± 0.00^c^	0.48 ± 0.01^b^	0.41 ± 0.01^c^
6	0.50 ± 0.01^a^	0.39 ± 0.01^b^	0.36 ± 0.01^b^	0.40 ± 0.00^b^	0.38 ± 0.02^b^	0.42 ± 0.02^b^	0.38 ± 0.01^b^
8	0.49 ± 0.01^a^	0.38 ± 0.01b	0.34 ± 0.01^b^	0.39 ± 0.01^b^	0.37 ± 0.00^b^	0.38 ± 0.01^b^	0.36 ± 0.01^b^
10	0.48 ± 0.01^a^	0.37 ± 0.01^b^	0.30 ± 0.00^b^	0.38 ± 0.01^b^	0.37 ± 0.01^b^	0.38 ± 0.02^b^	0.34 ± 0.02^b^
12	0.49 ± 0.01^a^	0.35 ± 0.02^b^	0.28 ± 0.01^b^	0.36 ± 0.01^b^	0.36 ± 0.01^b^	0.37 ± 0.01^b^	0.33 ± 0.01^b^
24	0.51 ± 0.01^a^	0.33 ± 0.00^b^		0.35 ± 0.02^b^	0.34 ± 0.01^b^	0.36 ± 0.01^b^	0.32 ± 0.01^b^
36	0.51 ± 0.00^a^	0.32 ± 0.01^b^		0.35 ± 0.01^b^	0.33 ± 0.01^b^	0.34 ± 0.02^b^	0.31 ± 0.01^b^
48	0.49 ± 0.02^a^	0.31 ± 0.01^b^		0.33 ± 0.01^b^	0.31 ± 0.01^b^	0.32 ± 0.00^b^	0.31 ± 0.02^b^
60	0.48 ± 0.01^a^	0.30 ± 0.00^b^		0.32 ± 0.01^b^	0.31 ± 0.02^b^	0.30 ± 0.00^b^	0.29 ± 0.01^b^
72	0.53 ± 0.00a	0.30 ± 0.00^b^		0.30 ± 0.00^b^	0.28 ± 0.01^b^	0.28 ± 0.01^b^	0.28 ± 0.02^b^
120	0.51 ± 0.01^a^	0.29 ± 0.01^b^		0.30 ± 0.01^b^	0.26 ± 0.01^b^	0.26 ± 0.01^b^	
168	0.50 ± 0.01^a^	0.28 ± 0.01^b^		0.28 ± 0.01^b^	0.25 ± 0.01^b^	0.25 ± 0.01^b^	
216	0.52 ± 0.01^a^	0.28 ± 0.02^b^		0.27 ± 0.01^b^	0.23 ± 0.0115^b^	0.24 ± 0.02^b^	
264	0.50 ± 0.00^a^	0.27 ± 0.01^b^		0.25 ± 0.02^b^	0.22 ± 0.01^b^	0.22 ± 0.00^b^	
312	0.51 ± 0.01^a^	0.26 ± 0.01^b^		0.24 ± 0.01^b^	0.22 ± 0.01^b^	0.22 ± 0.01^b^	
360	0.50 ± 0.00^a^	0.24 ± 0.003^b^		0.24 ± 0.01 ^b^	0.21 ± 0.01^b^	0.22 ± 0.01^b^	
408	0.49 ± 0.01^a^	0.22 ± 0.01^b^		0.22 ± 0.01^b^	0.21 ± 0.003^b^	0.21 ± 0.003^b^	
456	0.50 ± 0.01^a^	0.23 ± 0.01^b^		0.22 ± 0.01^b^	0.22 ± 0.01^b^	0.20 ± 0.003^b^	
504	0.50 ± 0.01^a^	0.23 ± 0.01^b^		0.22 ± 0.01^bc^	0.20 ± 0.00^c^	0.2 ± 0.01^bc^	

All values are expressed as mean ± SE; a, b, c, d, e, f, g, values with superscript within rows are significantly different at *P* < 0.05

### Estradiol concentration

The changes in mean concentration of EST in all groups are summarized in Table 5. After 6, 8, 10 and 12 h of inoculation, EST concentration was lower (*p* < 0.05) in G3 when compared to other inoculation groups. After 24 h, EST concentration was lower (*p* < 0.05) in all inoculation groups in comparison to the control. Similarly, EST concentration decreased gradually in all inoculation groups as time elapsed.

**Table 5:** Changes in mean concentration of Estradiol hormone in prepubertal buffalo heifers following inoculation with *Pasteurella multocida* B: 2 and its immunogens (LPS and OMP)

Time (hr)	Inoculated with phosphate buffer saline	Inoculated with *P.multocida* B:2		Inoculated with LPS extracted from *P.multocida* B:2		Inoculated with OMPs extracted from *P.multocida* B:2	
	Control –G1	Oral route -G2	Subcutaneous route - G3	Oral route- G4	Intravenous route – G5	Oral route - G 6	Subcutaneous route - G7
0	51.26 ± 0.56^a^	51.43 ± 0.00^a^	49.99 ± 0.45^ab^	48.91 ± 0.05^b^	50.72 ± 0.42^a^	49.19 ± 0.11^ab^	48.32 ± 0.58^b^
2	52.19 ± 0.02^a^	41.12 ± 0.01^d^	43.99 ± 0.01^bc^	43.57 ± 0.33^bc^	42.96 ± 0.02^cd^	41.13 ± 0.08^d^	44.32 ± 0.02^b^
4	48.16 ± 0.01^a^	40.65 ± 0.19^cd^	32.55 ± 0.51^d^	41.46 ± 0.01^c^	40.16 ± 0.58^cd^	40.46 ± 0.27^cd^	42.61 ± 0.03^b^
6	50.01 ± 0.00^a^	39.51 ± 0.00^b^	30.31 ± 0.18^f^	38.74 ± 0.15^d^	39.07 ± 0.00^c^	39.07 ± 0.00^c^	38.22 ± 0.052^e^
8	48.33 ± 0.58^a^	37.47 ± 0.64^ab^	27.76 ± 0.14^d^	36.18 ± 0.10^b^	38.11 ± 0.01^ab^	38.89 ± 0.51^ab^	35.24 ± 0.017^c^
10	48.11 ± 0.02^a^	36.21 ± 0.00^c^	26.66 ± 0.38^f^	34.98 ± 0.01^d^	37.76 ± 0.14^b^	35.31 ± 0.18^d^	29.7 ± 0.64^e^
12	50.31 ± 0.10^a^	32.57 ± 0.25^c^	20.33 ± 0.00^e^	34.72 ± 0.12^bc^	36.78 ± 0.13^b^	34.76 ± 0.44^bc^	29.85 ± 0.02^d^
24	51.22 ± 0.00^a^	31.19 ± 0.02^c^		33.91 ± 0.58^b^	32.84 ± 0.49^b^	30.06 ± 0.00^d^	28.34 ± 0.06^e^
36	48.41 ± 0.08^a^	30.88 ± 0.07^c^		33.02 ± 0.00^b^	30.35 ± 0.00^cd^	29.92 ± 0.53^d^	28.77 ± 0.13^d^
48	50.73 ± 0.00^a^	30.43 ± 0.58^bc^		31.33 ± 0.19^b^	30.12 ± 0.58^bcd^	27.18 ± 0.58^d^	27.99 ± 0.01^cd^
60	49.05 ± 0.58^a^	28.18 ± 0.02^bc^		30.93 ± 0.04^b^	28.17 ± 0.10^bcd^	26.14 ± 0.58^cd^	25.43 ± 0.10^d^
72	52.33 ± 0.06^a^	28.04 ± 0.00^d^		31.2 ± 0.5774^c^	36.78 ± 0.13^b^	24.41 ± 0.50^e^	22.17 ± 0.00^f^
120	49.13 ± 0.05^a^	27.59 ± 0.34^c^		28.37 ± 0.00^b^	26.34 ± 0.19^d^	23.96 ± 0.02^e^	
168	52.19 ± 0.04^a^	26.78 ± 0.13^b^		26.66 ± 0.38^b^	24.03 ± 0.00^b^	22.19 ± 0.00^c^	
216	51.64 ± 0.21^a^	26.33 ± 0.00^b^		26.31 ± 0.40^bc^	22.47 ± 0.31^bc^	22.35 ± 0.57^c^	
264	48.16 ± 0.58^a^	26.42 ± 0.66^b^		25.93 ± 0.54^bc^	22.15 ± 0.02^c^	22.49 ± 0.38^bc^	
312	48.41 ± 0.02^a^	24.98 ± 0.23^b^		24.06 ± 0.04^c^	21.14 ± 0.02^c^	22.01 ± 0.56^c^	
360	48.1 ± 0.58^a^	24.09 ± 0.18^b^		23.54 ± 0.27^bc^	20.94 ± 0.26^c^	20.21 ± 0.58^c^	
408	50.73 ± 0.16^a^	22.27 ± 0.15^b^		22.2 ± 0.318^bc^	20.91 ± 0.43^cd^	19.87 ± 0.50^d^	
456	50.2 ± 0.058^a^	20.17 ± 0.10^c^		21.88 ± 0.29^c^	20.66 ± 0.25^c^	19.43 ± 0.00^d^	
504	49.53 ± 0.38^a^	19.99 ± 0.01^bc^		21.09 ± 0.00^b^	20.03 ± 0.16^bc^	18.18 ± 0.10^c^	

All values are expressed as mean ± SE; a, b, c, d, e, f, g, values with superscript within rows are significantly different at *P* < 0.05

### Progesterone concentration

The changes in mean PG concentration in all groups are summarized in Table 6. The concentration of PG was comparable in all inoculation groups and control after 24 h. However, after 4 h, all inoculation groups had a lower (*p* < 0.05) PG concentration in comparison to the control. Similarly, PG concentration gradually declined in all inoculation groups as time elapsed.

**Table 6:** Changes in mean concentration of Progesterone hormone in Pre-pubertal buffalo heifers following inoculation with *Pasteurella multocida* B: 2 and its immunogens (LPS and OMP)

Time (hr)	Inoculated with PBS	Inoculated with *P.multocida* B:2		Inoculated with LPS extracted from *P.multocida* B:2		Inoculated with OMPs extracted from *P.multocida* B:2	
	Control G1	Oral route G2	Subcutaneous route - G3	Oral route G4	Intravenous route – G5	Oral route G 6	Subcutaneous route - G7
0	1.64±0.01^a^	1.68±0.17^a^	1.67±0.06^a^	1.66±0.19^a^	1.7±0.00^a^	1.64±0.00^a^	1.69±0.06^a^
2	1.70±0.017^a^	1.22±0.06^ab^	1.04±0.00^b^	1.22±0.01^ab^	1.34±0.07^ab^	1.32±0.12^ab^	1.12±0.00^b^
4	1.65±0.12^a^	0.9±0.00^e^	0.86±0.06^e^	1.12±0.00^c^	1.2±0.00^b^	1.13±0.01^c^	1.02±0.00^d^
6	1.65±0.01^a^	0.83±0.06^c^	0.67±0.10^c^	0.93±0.04^bc^	1.01±0.00^b^	0.96±0.02^bc^	0.93±0.01^bc^
8	1.69±0.17^a^	0.76±0.12^b^	0.54±0.01^b^	0.86±0.08^b^	0.92±0.01^b^	0.92±0.01^b^	0.89±0.06^b^
10	1.64±0.11^a^	0.73±0.01^cd^	0.48±0.00^e^	0.82±0.02^bc^	0.84±0.02^bc^	0.88±0.00^b^	0.66±0.04^d^
12	1.63±0.17^a^	0.64±0.01^b^	0.41±0.01^d^	0.78±0.06^b^	0.76±0.01^b^	0.79±0.06^b^	0.49±0.02^c^
24	1.68±0.06^a^	0.62±0.01^f^		0.66±0.01^c^	0.64±0.00^d^	0.74±0.01^b^	0.42±0.00^e^
36	1.67±0.01^a^	0.59±0.00^b^		0.61±0.01^b^	0.61±0.04^b^	0.71±0.00^c^	0.38±0.01^d^
48	1.68±0.1^a^	0.54±0.06^b^		0.59±0.04^b^	0.58±0.01^b^	0.55±0.01^b^	0.32±0.02^b^
60	1.68±0.17^a^	0.47±0.01^cd^		0.54±0.00^b^	0.53±0.00^c^	0.47±0.01^cd^	0.28±0.01^d^
72	1.69±0.11^a^	0.44±0.02^bcd^		0.51±0.02^b^	0.48±0.01^bc^	0.43±0.01^cd^	0.24±0.01^d^
120	1.63±0.01^a^	0.31±0.00^e^		0.52±0.01^b^	0.44±0.03^c^	0.36±0.01^d^	
168	1.64±0.12^a^	0.26±0.015^c^		0.48±0.01^b^	0.37±0.01^bc^	0.35±0.02^bc^	
216	1.7±0.12^a^	0.34±0.02^b^		0.36±0.02^b^	0.32±0.00^b^	0.32±0.01^b^	
264	1.64±0.01^a^	0.29±0.01^b^		0.33±0.01^b^	0.31±0.01^b^	0.3±0.00^b^	
312	1.65±0.01^a^	0.28±0.02^b^		0.33±0.01^b^	0.28±0.00^b^	0.29±0.01^b^	
360	1.66±0.12^a^	0.26±0.01^c^		0.33±0.01^b^	0.27±0.01^c^	0.3±0.018^c^	
408	1.68±0.12^a^	0.24±0.01^c^		0.29±0.012^b^	0.25±0.01^bc^	0.28±0.01b^c^	
456	1.7±0.06^a^	0.21±0.00^c^		0.27±0.01^bc^	0.24±0.01^b^	0.26±0.01b^c^	
504	1.67±0.03^a^	0.22±0.003^b^		0.23±0.01^b^	0.22±0.01^b^	0.23±0.01^b^	

All values are expressed as mean ± SE; a, b, c, d, e, f, g, values with superscript within rows are significantly different at *P* < 0

## Discussion

Previous study by Jesse et al. [9] in mouse models had shown the presence of both reproductive organ lesions and pituitary lesions in both male and female mice inoculated with *P. multocida* and its LPS. The lesions reported were both inflammatory and degenerative lesions in these organs with the *P. multocida* group showing more lesion severity and increased testosterone level than the LPS group. However, estrogen and progesterone in female mice were higher in the LPS group [9]. Similarly, a recent report by Ibrahim et al. [10, 11] showed evidence of reproductive pathological lesions characterized by degenerative and inflammatory changes in the ovaries, uterus, vagina, the uterine horns and supramammary glands of pre-pubertal buffalo heifers inoculated with *P. multocida* and its immunogens. In the study, lesion severity was more severity in the OMPs inoculation group than in the *P. multocida* and LPS groups [10, 11]. In this study, we observed histopathological changes in the anterior pituitary gland and these changes were more severe following inoculation of *P. multocida* B:2 subcutaneously and its OMPs both orally and subcutaneously. This is in agreement with the previous study by Jesse et al. [9] in mice, which showed pituitary gland lesions following *P.multocida* B:2 and LPS inoculation. However, the lesion severity in mice following oral inoculation of *P. multocida* B:2 was higher than what was observed in buffaloes in this study. This may be attributed to the body mass index in the buffaloes as well as differences in the digestive tract of the buffalo and the mice; whilst the inoculum goes directly into the stomach of the mice, in the buffalo, it passes through the fore stomach (rumen, reticulum and omasum) thus prolonging the time of absorption and incubation period. On the other hand the severity of lesions in the pituitary gland following inoculation of OMPs is similar to the severity of reproductive lesions observed in the buffaloe calves following OMPs inoculation [11]. This indicates that the bacterial OMPs is perhaps one of the most lethal components of *P. multocida* B:2 serotype.

In this study, we observed decreased hormonal concentrations of GnRH, LH, FSH, EST and PG in all the inoculation groups. This decline in the hormonal concentrations increased as the time of inoculation elapsed, indicating that the more the incubation period, the more the effect of the bacteria and immunogens on hormone production/secretion. The study by Jesse et al. [9] in mouse model showed an increased PG and decreased EST concentrations following oral inoculation of both *P. multocida* B:2 and its bacterial LPS. The difference in the PG concentration with buffaloes in this study may be attributed to the difference in cycling pattern between mice and buffalo, or due to the presence of an active corpus leutem in the mice, since they were not synchronized prior to the experimentation. The decline in the concentrations of reproductive hormones observed here can also be attributed to the lesions observed in the pituitary glands rather than to lesions reported in the reproductive tract [10, 11]. Since it was observed from this study that the most affected cells were the basophils which consists of the gonadotropes, lower levels of LH and FSH is expected as a result of the reversible (degeneration) or irreversible (necrosis) damage induced by *P. multocida* and its immunogens, especially the OMPs.

The pituitary gland is one of the most important endocrine glands in the body because it controls the activities of most other glands [14]. The different phases of the reproductive cycle are regulated by several sequential events and interactions between hypothalamic releasing hormones, hormones secreted from the pituitary and sex steroid hormones secreted by the ovary. Lack of integration, synchronization or endocrine imbalances at any phase of the sequence may result in reproductive insufficiency [15]. In the normal female animal, the hypothalamus secretes the GnRH which in turn causes the release of LH and FSH from the anterior pituitary. The FSH stimulates the follicles which grow and release estrogen. Progesterone on the other hand is produced by the corpus leutem within the ovaries [16]. Based on the above, *P. multocida* B:2 and its immunogens; OMPs and LPS were seen to suppress the hypothalamic production of GnRH, which in turn suppressed the production of LH and FSH produced by the anterior pituitary. Furthermore, a low plasma concentration of FSH and LH results in poor follicular development and reduced estrogen and progesterone production by the developing follicles. In an earlier study by Faccio et al. [17], the authors reported decreased concentrations of LH, FSH, PG and EST in female rats infected with *Trypanosoma evansi*. This was attributed to increased production of nitrite, advanced oxidation protein products (AOPP), and thiobarbituric acid reactive substances (TBARS) in the infected animals. Although the authors did not determine the levels of GnRH, they proposed the decreased hormonal activity to be associated with suppression of GnRH production by the hypothalamus due to oxidative damage. This assertion may be true since in this study, we observed decreased levels of GnRH in infected buffalo calves. Although Pasteurella and Trypanosoma have different mechanisms of pathogenesis, the nature of reproductive dysfunction may be similar in the two diseases. Other studies using different bacterial pathogens; *Corynebacterium pseudotuberculosis* and *Brucella mellitensis* reported variations in female reproductive hormones and pathological lesions in the female reproductive organs of mice and goats experimentally inoculated with the bacteria [12, 13, 18]. Based on previous studies by Chung et al. [19–21], the inoculation of *P. multocida* and immogens in buffalo calves resulted in different clinic-pathological changes in the lungs, trachea, heart, liver, spleen, kidney and submandibular lymph nodes. In the study, buffaloes from the *P. multocida* and OMPs subcutaneous inoculation groups showed more severe gross and histopathological lesions. Similarly, these inoculation groups showed significant changes in hematological and biochemical parameters. In a related study by Marza et al. [22], the inoculation of *P. multocida* subcutaneously to buffaloes resulted in marked histopathological changes in the brain of buffaloes. Furthermore, there was successful isolation of the bacteria from the brain. This finding is very important and pertinent to this study since the pituitary gland is attached to the brain through the hypophysis. Thus an infection of the brain will certainly extend to the pituitary gland. From this study, it can be said that animals that had survived HS may develop reproductive inefficiency which presents as infertility. This has not been previously reported in literature and is of immense significance in large animal production.

## Conclusion

This study reported for the first time the association between adenohypohyseal lesions, decreased hypothalamic production of GnRH and decreased hormonal levels of LH, FSH, EST and PG in buffalo heifers experimentally inoculated with *P. multocida* type B:2 and its immunogens; LPS and OMPs. Based on these findings, *P. multocida* and its immunogens can predispose to infertility in buffalo heifers.

## Abbreviations

EDTA: Ethylenediaminetetraacetic acid
EST: Estradiol
FSH: Follicle stimulating hormone
GnRH: Gonadotrophin releasing hormone
HRP: Horseradish peroxidase
HS: Hemorrhagic septicemia
LH: Leutienizing hormone
LPS: Lipopolysaccharide
OMPs: Outer membrane proteins
PG: Progesterone
